# Diagnostic value of gastric shake test for hyaline membrane disease in preterm infant 

**Published:** 2014-07

**Authors:** Mahmood NooriShadkam, Mohammad Hossein Lookzadeh, Mahmood Taghizadeh, Azam Golzar, Zahra NooriShadkam

**Affiliations:** 1 *Department of Pediatrics, Children Growth Disorder Research Center, Shahid Sadoughi University of Medical Sciences, Yazd, Iran.*; 2 *Children Growth Disorder Research Center, Shahid Sadoughi University of Medical Sciences, Yazd, Iran.*; 3 *Medical School, Shahid Sadoughi University of Medical Sciences, Yazd, Iran.*

**Keywords:** *Gastric aspirate shake test*, *Hyaline membrane disease*, *Neonate*, *Premature*, *Surfactant*

## Abstract

**Background:** Hyaline membrane disease (HMD) has remained a common neonatal problem and is a cause of morbidity in infants. The shake test can be used to assess whether surfactant is present in the infant's lungs at birth.

**Objective:** The goal of this study was to determine the usefulness and accuracy of gastric aspirate shake test for the diagnosis of two HMD.

**Materials and Methods: **This was a diagnostic accuracy study carried out on 49 preterm infant born at Shahid Sadoughi hospital in 2012 (25 newborns without pulmonary diseases and 24 newborns with HMD). Shortly after birth, the shake test was performed using gastric fluid. The results of the shake test were correlated with definitive diagnosis of HMD.

**Results: **All infants who developed HMD had negative test results. In 23 of 25 infants with no respiratory distress, the test was positive. Our findings indicated that the gastric aspirate shake test has 100% sensitivity, 92% specificity, a 92.3% predictive value for surfactant deficiency, and 100% predictive value for surfactant sufficiency.

**Conclusion:** According to this study gastric shake test (GST) is a reliable test and is a simple procedure to identify those neonates who will develop respiratory distress syndrome (RDS) and therefore to decide prophylactic exogenous surfactant replacement.

## Introduction

Respiratory distress syndrome (RDS) is the most common problem in neonatal nurseries, with an incidence that is inversely proportional to gestational age (GA) and birth weight (BW); 60-80% of infants with GA of <28 weeks and 30% of those with GA of 32-36 weeks develop RDS (1). Despite major advances in the understanding and management of respiratory distress in the newborn, hyaline membrane disease (HMD) has remained the most common cause of death and handicap in premature infants that is associated with a 30% mortality rate in the neonatal population (2, 3). 

Therefore an urgent work up and appropriate therapy seems to be essential. The risk for development of RDS increases with maternal diabetes, multiple births, cesarean delivery, precipitous delivery, asphyxia, cold stress, and a maternal history of previously affected infants. The lack of pulmonary surfactant results in progressive alveolar collapse, respiratory distress and, often, requires resuscitation at birth (especially with a birth weight <1000 g) (1). Characteristically, tachypnea, prominent (often audible) grunting, intercostals and sub costal retractions, nasal flaring, and cyanosis are noted. The clinical course, chest radiographic findings, and blood gas and acid-base values help establish the clinical diagnosis. Exogenous surfactant replacement is now an essential treatment for premature neonates with idiopathic RDS (4). 

The importance of identifying infants with HMD as soon as possible after delivery, to facilitate their early transfer to a neonatal intensive care unit, has been shown (5). A rapid, simple, and reproducible test of pulmonary maturity in the newborn infant at risk would therefore be of great value. 

Biochemical tests for measuring the lecithin to sphingomyelin (L/S) ratio and phosphatidylglycerol (PG) levels, or immunoassays for surfactant-associated proteins and other proteins require technical skill and are time consuming and expensive. So a common bedside test used for determining surfactant deficiency is the shake test (6-10). The purpose of this study was to evaluate the gastric aspirate shake test GST to rapidly and reliably identify surfactant assessment of neonates with Hyaline Membrane Disease.

## Materials and methods

We performed a cross sectional study in the neonatal intensive care unit of Shahid Sadoughi hospital in 2012. All preterm babies with gestational less than 34 weeks (calculated from the recorded date of mother’s last menstrual period (LMP) and in the absence of a known date of LMP, Ultrasound can be used during the third trimester) were enrolled. The neonates who had severe congenital anomalies or conditions incompatible with life, moderate and severe birth asphyxia, persistent pulmonary hypertension of the newborn (PPHN), meconium aspiration, pneumothorax were excluded. History of pregnancy was taken from medical records.

All babies were examined at birth and information regarding birth weight, height, head circumference, gestational age and mode of delivery were recorded. Radiological findings and Silverman Anderson retraction score after resuscitation were also recorded. Informed consent was obtained from the children's parents.

**Figure 1:**
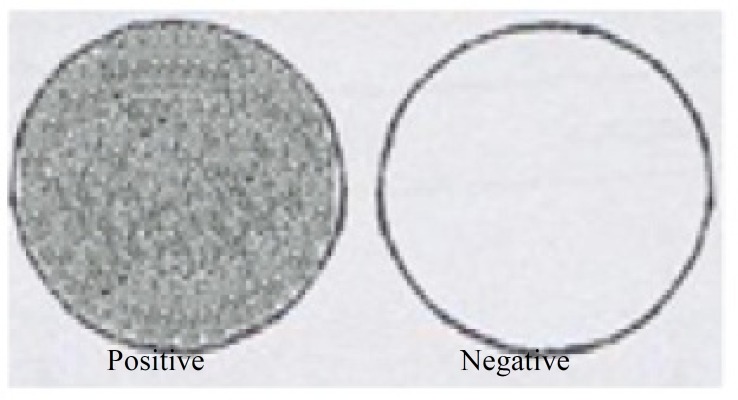
Schematic diagram showing interpretations of gastric aspirate shake test

From all babies 0.5 ml of gastric fluid was obtained within 20 min of birth then mixed with an equal volume of normal saline for15 sec; 1 ml of 95% ethanol was then added and the mixture agitated for 15 sec. After standing for 15 min, the air-liquid interface was examined for bubbles. If no bubbles are present then the test is NEGATIVE (very little surfactant is present). If bubbles are present right across the surface of the fluid, then the test is POSITIVE (adequate amounts of surfactant). (Figure 1). After the shake test the samples were divided into two groups: 1) The first group received surfactant as soon as possible based on hyaline membrane disease symptoms, Silverman Anderson retraction score>6, or radiological findings typical of RDS. 2) The second group of infants in the control group, which includes premature infants less than 34 weeks, without RDS and in no respiratory distress. 

The results of the tests were correlated with clinical data and outcome of disease. The Ethic Committee of Shahid Sadoughi University of Medical Sciences confirmed this study.

Statistical analysis

Data were analyzed using SPSS version 18. Student’s *t*-test analysis and chi-square test was used as appropriate. Differences were considered to be statistically significant when p<0.05.

## Results

During the period 51 preterm babies were admitted to the unit. Three infants were excluded because of refused parental consent or transferred to other hospitals (Two of these infants developed respiratory distress). We collected satisfactory gastric aspirates from 49 neonates within 20 min after birth. The infants met all the eligibility criteria, and the samples were used to perform the shake test. The differences in clinical characteristic between premature neonates who did (n= 24) and did not (n= 25) develop RDS are given in table I. 

Out of the 49 babies enrolled in the study, 26 were males and 23 females (p=0.571). Mean birth weight was 1193±245 g and mean gestational age 29.5 weeks (range 28-31 weeks) in treatment group. There was no significant difference in gender, mode of delivery and birth head circumference between the groups. Mean height (p<0.001), weight (p<0.001) and gestational age (p=0.015) were significantly lower in treatment group compared to control group. 24 infants of Group 1 had radiological findings of RDS in contrast to 5 of Group 2 (p<0.001). There was a significantly higher Silverman Anderson retraction score in Group 1 compared with group 2 (p<0.01) (Table II). 

It can be seen that a negative shake test has included all infants developing respiratory symptoms (sensitivity 100%) and that in this group 24 of the 26 infants develop respiratory symptoms (specificity 92%). The probability of RDS with a positive predictive value was 92.3%; the probability of no RDS when the test was negative (negative predictive value) was 100%. In our control group of 25 preterm infants we found 8% incidence of false-negative results. The relationship between the shake test result and the clinical diagnosis is shown in Table III.

**Table I:** Characteristics of the studied neonates

**Characteristics**	**RDS (Group 1)**	**No respiratory distress (Group 2)**	**p-value**
Male (%)	50	50	0.571
Birth weight (mean ± SD) (gr)	1193 ± 245	1547 ± 160	<0.001
Birth height (mean ± SD) (cm)	42.6 ± 1.6	44.9 ± 1.44	<0.001
Birth head circumference (mean ± SD) (cm)	30.75 ± 1.9	32.5 ± 0.71	0.095
Gestational age (mean ± SD) (weeks)	29.5 ± 1.5	31.9 ± 0.8	0.015
Cesarean section (%)	84%	64%	0.089

RDS: Respiratory distress syndrome

* Independent t-test and chi-square

**Table II:** Radiological findings of the studied neonates

** Radiological findings**	**No finding**	**Mild**	**Moderate**	**Severe**
**Groups**				
RDS Group 1 (n, %)	0	2 (8.3%)	5 (20.8%)	17 (70.83%)
No Respiratory Distress Group 2 (n, %)	20 (80%)	2 (8%)	3 (12%)	0

* The used statistical test: Chi-square test.

Comparing with control group, p <0.01

**Tables III:** Results of the shake test

** Groups**	**RDS (Group 1)**	**No respiratory distress (Group 2)**
**Result**		
Negative (n, %)	24 (100%)	2 (8%)
Positive (n, %)	0	23 (92%)
Total (n)	24	25

## Discussion

Our findings indicated that all infants who developed HMD had negative test result. We also showed specificity of 92%, positive predictive value of 92.3% and negative predictive value of 100% for developing HMD. RDS continues to be a major determinant in the timing of delivery, which in developed countries is a major contributor to neonatal morbidity and mortality. Over 30 years ago Gluck and colleagues predicted the presence or absence of RDS in the newborn infants by measuring the amniotic fluid lecithin: sphingomyelin (L: S) ratio (11, 12). 

Although it is remained a gold standard today, but it is not routinely available, particularly in developing countries. The need for a rapid test prompted numerous assays of the functional and physical characteristics of amniotic fluid such as the ‘shake test’ and ‘tap test’ (8, 13). The GST was the first clinical postnatal test to identify immaturity of lungs (14). This study supports previous observations in that it confirms the usefulness of the GST in predicting the development of RDS (12, 15-19). 

In our study all infants who developed HMD had negative GST indicating surfactant deficiency, sensitivity and negative predictive value of 100%, but 8% of preterm infants with a negative test did not develop HMD because the sample included all preterm infants regardless of whether they had symptoms of respiratory distress. In a study by Chaudhari all 21 babies with a negative GST indicating surfactant deficiency developed HMD, a positive predictive value of 100%. However, they undertook GST only in infants who developed respiratory distress. They did not include a control group without respiratory distress as, in a pilot study; none of these babies had a negative GST (14). 

Similar to our study Mohammadi *et al* evaluated the GST and showed that all infants who developed HMD had negative or intermediate test results. The sensitivity and specificity of the GST for prediction of surfactant requirement in HMD patients were 100% and 64.8%, respectively, with a positive and negative predictive value of 62.5% and 100%, respectively (16). Tanswell evaluated a single-step shake test in all preterm infants and showed that 34% of them with a negative test did not develop HMD (20).

Pena-Camarena and Caballero-Zavaleta showed that the shake test had 97.5% sensitivity, 77.1% specificity, 70.9% predictive value for surfactant deficiency and 98.2% predictive value for surfactant sufficiency (21). Amoa *et al* found a sensitivity of 40%, a specificity of 95%, a positive predictive value of 63% and a negative predictive value of 88% (22). Gupta e*t al *found that GST had a predictive value of 96% for HMD and the test was comparable to the LS ratio of pharyngeal aspirate (23). Singh showed a sensitivity of 94.5% and specificity of 71% and Parekh *et al* found a predictive value of 92.3% (11, 24, 25). 

In contrast to our study several study confirmed the inaccuracy of the shake test in predicting RDS as reflected by a large number of false-positive results (26-28). However, our study showed that there were no false-positive results. Chaudhari *et al* found that babies in the positive and intermediate GST groups who did not develop HMD had respiratory problems such as transient tachypnoea of the newborn (TTNB), pneumonia and other respiratory diseases but we excluded the neonates who had moderate and severe birth asphyxia, PPHN, meconium aspiration, Pneumothorax (14). 

The results from this study and previous studies indicate that the gastric aspirate shake test can play role in assessing lung maturity in preterm infants before any clinical symptoms; this method enhances the simplicity of the test while retaining its reliability. Therefore, we suggest that this test be performed immediately after birth to identify high-risk neonates eligible for prophylactic treatment. This protocol would prevent unnecessary use of a very costly drug, even if it has few side effects.

## Footenote

***This article is extracted from M.D. thesis. (Mahmood Taghizadeh)***
